# *Angiostrongylus cantonensis* Eosinophilic Meningitis in an Infant, Tennessee, USA

**DOI:** 10.3201/eid2310.170978

**Published:** 2017-10

**Authors:** Tim Flerlage, Yvonne Qvarnstrom, John Noh, John P. Devincenzo, Arshia Madni, Bindiya Bagga, Nicholas D. Hysmith

**Affiliations:** St. Jude Children’s Research Hospital, Memphis (T. Flerlage);; University of Tennessee Health Science Center, Memphis, Tennessee, USA (T. Flerlage, J.P. Devincenzo, A. Madni, B. Bagga, N.D. Hysmith);; Centers for Disease Control and Prevention, Atlanta, Georgia, USA (Y. Qvarnstrom, J. Noh);; Le Bonheur Children’s Hospital, Memphis (J.P. Devincenzo, A. Madni, B. Bagga, N.D. Hysmith)

**Keywords:** *Angiostrongylus cantonensis*, meningitis/encephalitis, eosinophil-predominant pleocytosis, mild hypoglycorrhacia, rat lungworm, parasites, Tennessee, United States

## Abstract

*Angiostrongylus cantonensis*, the rat lungworm, is the most common infectious cause of eosinophilic meningoencephalitis worldwide. This parasite is endemic to Southeast Asia and the Pacific Islands, and its global distribution is increasing. We report *A. cantonensis* meningoencephalitis in a 12-month-old boy in Tennessee, USA, who had not traveled outside of southwestern Tennessee or northwestern Mississippi.

In 2016, a 12-month-old, fully vaccinated boy was admitted to a hospital in Memphis, Tennessee, USA, for evaluation of 18 days of daily fever, irritability, decreased oral intake, and emesis. His medical history was unremarkable, and he had no known contact with sick persons. He had not traveled outside the area comprising southwestern Tennessee and northwestern Mississippi. He lived in a nonagricultural rural area and was exposed to a vaccinated family dog. Wild rats had been observed in and around the home, and rat droppings had been found in the child’s bed. Raccoons were seen on the property; however, contact, either direct or through fomites such as latrines, was not reported. During a 17-day period, 2 evaluations by his primary care physician and 4 emergency department visits resulted in the diagnosis of fever of unknown origin and inpatient admission.

A cerebrospinal fluid (CSF) sample taken by lumbar puncture on day 20 of illness showed eosinophil-predominant pleocytosis, mild hypoglycorrhacia, and a mildly elevated protein level (Table). Magnetic resonance imaging of the brain and spine showed scattered areas of restricted diffusion throughout the brain parenchyma, leptomeningeal enhancement, and multifocal nodular enhancement along the ventral portion of multiple spinal levels. Serologic testing was negative for *Toxocara canis/cati*, *Strongyloides stercoralis*, *Ehrlichia chaffeenis, Rickettsia rickettsiae*, Epstein-Barr virus, HIV, and *Toxoplasma gondii*; a rapid plasma reagin was also negative. Tuberculin skin testing was negative. Results of CSF PCR for *Streptococcus pneumoniae*, herpes simplex virus, and enteroviruses were negative; CSF cryptococcal antigen testing was also negative. Due to concern for infection with *Baylisascaris procyonis*, the raccoon roundworm, physicians prescribed albendazole and dexamethasone. The patient’s temperature returned to normal, and his symptoms resolved. Upon discharge, he was to complete 3 weeks of albendazole and tapering doses of corticosteroids. Attending physicians repeated lumbar punctures on days 28, 41, and 56 (Table). 

**Table:** Results of cerebrospinal fluid testing for 12-month-old boy with meningoencephalitis, Memphis, Tennessee, USA, 2016*

Test	Reference range	Day of illness			
		20	28	41	56
Leukocytes, cells/mm^3^	0–8	547	55	2,979	115
Polymorphonuclear cells, %	0–1	0	4	20	4
Lymphocytes, %	0–5	44	80	65	64
Monocytes, %	0–5	20	6	10	21
Eosinophils, %	NA	36	10	5	11
Erythrocytes	<0	0	14	0	45
Glucose, mg/dL	40–70	37	25	22	27
Protein, mg/dL	15–45	67	74	164	104
Gram stain	NA	Few leukocytes; 0 organisms	Rare leukocytes; rare erythrocytes; 0 organisms	Moderate leukocytes; rare erythrocytes; 0 organisms	Rare leukocytes; 0 organisms
Bacterial culture	NA	Sterile	Sterile	Sterile	Sterile

*NA, not applicable.

Physicians sent samples (CSF and serum) taken on day 20 to the Centers for Disease Control and Prevention (Atlanta, GA, USA) to test for *B. procyonis* roundworms and samples taken on day 56 to test for *Angiostrongylus cantonensis*, the rat lungworm. Results were negative for *B. procyonis* but positive for *A. cantonensis*. In addition, serum samples obtained at the time of the initial lumbar puncture were positive for *A. cantonensis* antibodies by investigational whole-worm Western blot.

The first documented human infection with *A. cantonensis* worms occurred in 1944 in Taiwan. Since then, >2,800 cases among humans have been reported; most have been in Southeast Asia and the Pacific islands (*1**;* Technical Appendix). In the late 1950s, the first report of human *A. cantonensis* infection in the United States occurred in Hawaii. *A. cantonensis* worms have since become endemic to wide-ranging tropical and subtropical locales in the Western Hemisphere, including the Hawaiian Islands (*2*), the Caribbean Islands (*3*), and South America (*4*).

The first report of the rat lungworm in the continental United States was in 1987, when Kim et al. found that 18% of rats sampled on necropsy in New Orleans, Louisiana, were infected with the nematode (*5*). First-stage *A. cantonensis* larvae from these rats produced infections in native gastropods, providing the potential for these parasites to become endemic to the region. A report ≈15 years later documented infection in vertebrates not only in New Orleans but also in other areas of Louisiana and Mississippi. *A. cantonensis* worms are now considered to be endemic to Louisiana (*5*). Infection has since been documented in rats (*6*), gastropods (*7*), and vertebrates (*8*) across a large area of the southern United States, from Oklahoma (*6*) to Florida (*7*,*8*).

Soon after the initial recognition in local animal reservoirs, the first reported *A. cantonensis* infection in a human acquired in the continental United States occurred in an 11-year-old boy residing in New Orleans. Since then, 3 additional cases have been reported in an 11-month-old, a 12-month-old, and a 19-month-old, all of whom resided in Houston, Texas, and had not traveled (*9*).

*A. cantonensis* infection causes a self-limited illness in which headaches, nonfocal neurologic findings, and cranial nerve involvement are the most common signs and symptoms. Optimal therapy has not been clearly defined, and symptomatic management is an option for this self-limited illness. When therapy is prescribed, corticosteroids alone or in combination with antihelminth medications are most commonly used. In a prospective study that followed up on 3 previous studies, Chotmongkol et al. confirmed that a 2-week course of corticosteroids shortened the duration of headache and reduced the need for repeated lumbar puncture (*10*). The study concluded that corticosteroids plus albendazole was no better than corticosteroids alone.

International shipping and the ability of *A. cantonensis* worms to use diverse species of gastropods as intermediate hosts have all contributed to this parasite becoming a pathogen of increasing public health concern (*5*). Angiostrongyliasis should be considered in the differential diagnosis of prolonged fever of unknown origin with compatible clinical and laboratory findings.

Technical Appendix Additional reports of human infection with *Angiostrongylus cantonensis*, the rat lungworm.
