# Supplementary material for: Angiostrongylus cantonensis Eosinophilic Meningitis in an Infant, Tennessee, USA
Source: Emerg Infect Dis. 2017 Oct;23(10):1756–8. doi: 10.3201/eid2310.170978 (PMC5621543; doi:10.3201/eid2310.170978)
Supplement: Technical Appendix — Additional reports of human infection with Angiostrongylus cantonensis, the rat lungworm. [file 17-0978-Techapp-s1.pdf]

Article DOI: https://doi.org/10.3201/eid2310.170978

Angiostrongylus cantonensis Eosinophilic Meningitis in an Infant, Tennessee, USA

Technical Appendix

Additional reports of human infection with *Angiostrongylus cantonensis*, the rat lungworm.

1. Chen HT. A new pulmonary nematode of rats, *Pulmonema cantonensis* ng, nsp from Canton. Ann Parasitol. 1935;13:312–7.

2. Wang Q-P, Lai D-H, Zhu X-Q, Chen X-G, Lun Z-R. Human angiostrongyliasis. Lancet Infect Dis. 2008;8:621–30. PubMed http://dx.doi.org/10.1016/S1473-3099(08)70229-9

3. Wang Q-P, Wu Z-D, Wei J, Owen RL, Lun Z-R. Human *Angiostrongylus cantonensis*: an update. Eur J Clin Microbiol Infect Dis. 2012;31:389–95. PubMed http://dx.doi.org/10.1007/s10096-011-1328-5

4. Graeff-Teixeira C, da Silva AC, Yoshimura K. Update on eosinophilic meningoencephalitis and its clinical relevance. Clin Microbiol Rev. 2009;22:322–48. PubMed http://dx.doi.org/10.1128/CMR.00044-08

5. Nomura S, Lin PH. First case report of human infection with *Haemostrongylus ratti*, Yokagawa. Taiwan No Ikai. 1945;3:589–92.

6. Kliks MM, Palumbo NE. Eosinophilic meningitis beyond the Pacific Basin: the global dispersal of a peridomestic zoonosis caused by *Angiostrongylus cantonensis*, the nematode lungworm of rats. Soc Sci Med. 1992;34:199–212. PubMed http://dx.doi.org/10.1016/0277-9536(92)90097-A

7. Kim JR, Hayes KA, Yeung NW, Cowie RH. Diverse gastropod hosts of *Angiostrongylus cantonensis*, the rat lungworm, globally and with a focus on the Hawaiian Islands. PLoS One. 2014;9:e94969. PubMed http://dx.doi.org/10.1371/journal.pone.0094969

8. Barratt J, Chan D, Sandaradura I, Malik R, Spielman D, Lee R, et al. *Angiostrongylus cantonensis*: a review of its distribution, molecular biology and clinical significance as a human pathogen. Parasitology. 2016;143:1087–118. PubMed http://dx.doi.org/10.1017/S0031182016000652

9. Campbell BG, Little MD. The finding of *Angiostrongylus cantonensis* in rats in New Orleans. Am J Trop Med Hyg. 1988;38:568–73. PubMed http://dx.doi.org/10.4269/ajtmh.1988.38.568

10. Kim DY, Stewart TB, Bauer RW, Mitchell M. *Parastrongylus* (=*Angiostrongylus*) *cantonensis* now endemic in Louisiana wildlife. J Parasitol. 2002;88:1024–6. PubMed http://dx.doi.org/10.1645/0022-3395(2002)088[1024:PACNEI]2.0.CO;2

11. York EM, Creecy JP, Lord WD, Caire W. Geographic range expansion for rat lungworm in North America. Emerg Infect Dis. 2015;21:1234–6. PubMed http://dx.doi.org/10.3201/eid2107.141980

12. Rosen L, Chappell R, Laqueur GL, Wallace GD, Weinstein PP. Eosinophilic meningoencephalitis caused by a metastrongylid lung-worm of rats. JAMA. 1962;179:620–4. PubMed http://dx.doi.org/10.1001/jama.1962.03050080032007

13. Qvarnstrom Y, Bishop HS, da Silva AJ. Detection of rat lungworm in intermediate, definitive, and paratenic hosts obtained from environmental sources. Hawaii J Med Public Health. 2013;72(Suppl 2):63–9. PubMed

14. Hochberg NS, Park SY, Blackburn BG, Sejvar JJ, Gaynor K, Chung H, et al. Distribution of eosinophilic meningitis cases attributable to *Angiostrongylus cantonensis*, Hawaii. Emerg Infect Dis. 2007;13:1675–80. PubMed http://dx.doi.org/10.3201/eid1311.070367

15. Hochberg NS, Blackburn BG, Park SY, Sejvar JJ, Effler PV, Herwaldt BL. Eosinophilic meningitis attributable to *Angiostrongylus cantonensis* infection in Hawaii: clinical characteristics and potential exposures. Am J Trop Med Hyg. 2011;85:685–90. PubMed http://dx.doi.org/10.4269/ajtmh.2011.11-0322

16. Thyssen A, Mitchell M, Qvarnstrom Y, Rao S, Benke TA, Glodé MP. Eosinophilic meningitis in a previously healthy 13-year-old child. Pediatr Infect Dis J. 2013;32:194, 198. PubMed http://dx.doi.org/10.1097/INF.0b013e31827c9726

17. Waugh CA, Lindo JF, Lorenzo-Morales J, Robinson RD. An epidemiological study of *A. cantonensis* in Jamaica subsequent to an outbreak of human cases of eosinophilic meningitis in 2000. Parasitology. 2016;143:1211–7. PubMed http://dx.doi.org/10.1017/S0031182016000640

18. Simoes RO, Monteiro FA, Sanchez E, Thiengo SC, Garcia JS, Costa-Neto SF, et al. Endemic angiostrongyliasis, Rio de Janeiro, Brazil. Emerg Infect Dis. 2011;17:1331–3. PubMed http://dx.doi.org/10.3201/eid1707.101822

19. Moreira VLC, Giese EG, Melo FTV, Simões RO, Thiengo SC, Maldonado A Jr, et al. Endemic angiostrongyliasis in the Brazilian Amazon: natural parasitism of *Angiostrongylus cantonensis* in

Rattus rattus and *R. norvegicus*, and sympatric giant African land snails, *Achatina fulica.* Acta Trop. 2013;125:90–7. PubMed http://dx.doi.org/10.1016/j.actatropica.2012.10.001

20. Iwanowicz DD, Sanders LR, Schill WB, Xayavong MV, da Silva AJ, Qvarnstrom Y, et al. Spread of the rat lungworm (*Angiostrongylus cantonensis*) in giant African land snails (*Lissachatina fulica*) in Florida, USA. J Wildl Dis. 2015;51:749–53. PubMed http://dx.doi.org/10.7589/2014-06-160
21. Stockdale-Walden HD, Slapcinsky J, Qvarnstrom Y, McIntosh A, Bishop HS, Rosseland B. *Angiostrongylus cantonensis* in introduced gastropods in Southern Florida. J Parasitol. 2015;101:156–9. PubMed http://dx.doi.org/10.1645/14-553.1
22. Duffy MS, Miller CL, Kinsella JM, de Lahunta A. *Parastrongylus cantonensis* in a nonhuman primate, Florida. Emerg Infect Dis. 2004;10:2207–10. PubMed http://dx.doi.org/10.3201/eid1012.040319
23. Kottwitz JJ, Perry KK, Rose HH, Hendrix CM. *Angiostrongylus cantonensis* infection in captive Geoffroy’s tamarins (*Saguinus geoffroyi*). J Am Vet Med Assoc. 2014;245:821–7. PubMed http://dx.doi.org/10.2460/javma.245.7.821
24. New D, Little MD, Cross J. *Angiostrongylus cantonensis* infection from eating raw snails. N Engl J Med. 1995;332:1105–6. PubMed http://dx.doi.org/10.1056/NEJM199504203321619
25. Foster CE, Nicholson EG, Chun AC, Gharfeh M, Anvari S, Seeborg FO, et al. *Angiostrongylus cantonensis* infection: a cause of fever of unknown origin in pediatric patients. Clin Infect Dis. 2016;63:1475–8. PubMed http://dx.doi.org/10.1093/cid/ciw606
26. Tsai HC, Liu YC, Kunin CM, Lee SS, Chen YS, Lin HH, et al. Eosinophilic meningitis caused by *Angiostrongylus cantonensis*: report of 17 cases. Am J Med. 2001;111:109–14. PubMed http://dx.doi.org/10.1016/S0002-9343(01)00766-5
27. Sawanyawisuth K, Chindaprasirt J, Senthong V, Limpawattana P, Auvichayapat N, Tassniyom S, et al. Clinical manifestations of Eosinophilic meningitis due to infection with *Angiostrongylus cantonensis* in children. Korean J Parasitol. 2013;51:735–8. PubMed http://dx.doi.org/10.3347/kjp.2013.51.6.735
28. Evans-Gilbert T, Lindo JF, Henry S, Brown P, Christie CD. Severe eosinophilic meningitis owing to *Angiostrongylus cantonensis* in young Jamaican children: case report and literature review. Paediatr Int Child Health. 2014;34:148–52. PubMed http://dx.doi.org/10.1179/2046905513Y.0000000106

29. Chen XG, Li H, Lun ZR. Angiostrongyliasis, Mainland China. Emerg Infect Dis. 2005;11:1645–7. PubMed http://dx.doi.org/10.3201/eid1110.041338

30. Morassutti AL, Rascoe LN, Handali S, da Silva AJ, Wilkins PP, Graeff-Teixeira C. Cross-reactivity of the 31 kDa antigen of *Angiostrongylus cantonensis* – Dealing with the immunodiagnosis of meningoencephalitis. Parasitology. 2017;144:459–63. PubMed

31. Qvarnstrom Y, Xayavong M, da Silva AC, Park SY, Whelen AC, Calimlim PS, et al. Real-Time Polymerase Chain Reaction Detection of *Angiostrongylus cantonensis* DNA in Cerebrospinal Fluid from Patients with Eosinophilic Meningitis. Am J Trop Med Hyg. 2016;94:176–81. PubMed http://dx.doi.org/10.4269/ajtmh.15-0146

32. Punyagupta S, Juttijudata P, Bunnag T. Eosinophilic meningitis in Thailand. Clinical studies of 484 typical cases probably caused by *Angiostrongylus cantonensis*. Am J Trop Med Hyg. 1975;24:921–31. PubMed http://dx.doi.org/10.4269/ajtmh.1975.24.921

33. Yii CY. Clinical observations on eosinophilic meningitis and meningoencephalitis caused by *Angiostrongylus cantonensis* on Taiwan. Am J Trop Med Hyg. 1976;25:233–49. PubMed http://dx.doi.org/10.4269/ajtmh.1976.25.233

34. Jin E, Ma D, Liang Y, Ji A, Gan S. MRI findings of eosinophilic myelomeningoencephalitis due to *Angiostrongylus cantonensis*. Clin Radiol. 2005;60:242–50. PubMed http://dx.doi.org/10.1016/j.crad.2004.05.012

35. Chotmongkol V, Sawanyawisuth K, Thavornpitak Y. Corticosteroid treatment of eosinophilic meningitis. Clin Infect Dis. 2000;31:660–2. PubMed http://dx.doi.org/10.1086/314036

36. Chotmongkol V, Wongjitrat C, Sawadpanit K, Sawanyawisuth K. Treatment of eosinophilic meningitis with a combination of albendazole and corticosteroid. Southeast Asian J Trop Med Public Health. 2004;35:172–4. PubMed

37. Chotmongkol V, Sawadpanitch K, Sawanyawisuth K, Louhawilai S, Limpawattana P. Treatment of eosinophilic meningitis with a combination of prednisolone and mebendazole. Am J Trop Med Hyg. 2006;74:1122–4. PubMed

38. Chotmongkol V, Kittimongkolma S, Niwattayakul K, Intapan PM, Thavornpitak Y. Comparison of prednisolone plus albendazole with prednisolone alone for treatment of patients with eosinophilic meningitis. Am J Trop Med Hyg. 2009;81:443–5. PubMed
